# Management and Prognosis of Heart Failure in Octogenarians: Final Report from the KorAHF Registry

**DOI:** 10.3390/jcm9020501

**Published:** 2020-02-12

**Authors:** Gyu Chul Oh, Hyun-Jai Cho, Sang Eun Lee, Min-Seok Kim, Jae-Joong Kim, Jin-Oh Choi, Eun-Seok Jeon, Kyung-Kuk Hwang, Shung Chull Chae, Sang Hong Baek, Seok-Min Kang, Byung-Su Yoo, Dong-Ju Choi, Youngkeun Ahn, Kye Hun Kim, Myeong-Chan Cho, Byung-Hee Oh, Hae-Young Lee

**Affiliations:** 1 Department of Internal Medicine, Seoul National University Hospital, Seoul 03080, Korea; david.gyuchul@gmail.com (G.C.O.); hyunjaicho@snu.ac.kr (H.-J.C.); ohbhmed@snu.ac.kr (B.-H.O.); 2 Department of Cardiology, Asan Medical Center, Seoul 05505, Korea; sangeunlee.md@gmail.com (S.E.L.); guess124@gmail.com (M.-S.K.); jjkim@amc.seoul.kr (J.-J.K.); 3 Department of Medicine, Sungkyunkwan University College of Medicine, Seoul 16419, Korea; choijean5@gmail.com (J.-O.C.);; 4 Department of Internal Medicine, Chungbuk National University College of Medicine, Cheongju 28644, Korea; kyungkukhwang@gmail.com (K.-K.H.); mccho@chungbuk.ac.kr (M.-C.C.); 5 Department of Internal Medicine, Kyungpook National University College of Medicine, Daegu 37224, Korea; scchae@knu.ac.kr; 6 Department of Internal Medicine, The Catholic University of Korea, Seoul 06591, Korea; whitesh@catholic.ac.kr; 7 Department of Internal Medicine, Yonsei University College of Medicine, Seoul 03722, Korea; smkang@yumc.yonsei.ac.kr; 8 Department of Internal Medicine, Yonsei University Wonju College of Medicine, Wonju 26426, Korea; yubs@yonsei.ac.kr; 9 Department of Internal Medicine, Seoul National University Bundang Hospital, Seongnam 13620, Korea; 10 Heart Research Center of Chonnam National University, Gwangju 61469, Korea; cecilyk@chonnam.ac.kr (Y.A.); cvkimkh@gmail.com (K.H.K.)

**Keywords:** heart failure, guideline-directed medical therapy, sex difference, octogenarians, J-curve, mortality

## Abstract

Treatment of heart failure (HF) in the elderly face many difficulties due to lack of robust evidence. We analyzed the outcome of HF in octogenarians using a nationwide HF registry. Among 5625 patients from the Korean Acute Heart Failure (KorAHF) registry, prognosis of octogenarian HF and the association of guideline-directed medical therapy (GDMT) with mortality and readmissions were analyzed. Octogenarian patients (1185, 22.4%) showed a higher mortality, and males were especially at increased risk (HR (hazard ratio) 1.19, 95% CI 1.01–1.40). A J-curve association between blood pressure (BP) and mortality was observed regardless of age, but the nadir value was lower in octogenarians (123.8 vs. 127.9 mmHg for systolic blood pressure (SBP); 67.1 vs. 73.9 mmHg for diastolic blood pressure (DBP), *p* < 0.001). Use of GDMT in octogenarian patients with HF and reduced ejection fraction (EF) were inadequate (74.3%, 47.1%, and 46.1% in octogenarians vs. 78.4%, 59.8%, and 55.2% in non-elderly for renin-angiotensin system inhibitors, beta-blockers, and aldosterone antagonists, respectively; all *p* < 0.05). However, those on medications had a significant reduction in 6 month mortality. For octogenarians with HF and preserved EF, angiotensin receptor blocker use reduced hospitalizations for HF in men (HR 0.19, 95% CI 0.04–0.87), but not in women (*p*-interaction = 0.037). HF in octogenarians were found to have different characteristics compared with the non-elderly. However, adequate use of GDMT was still associated with improved survival, and more attention should be given to prescribing medications with clinical benefits.

## 1. Introduction

In recent years, there has been a steep rise in global life expectancy, and more countries are expected to enter “aged” and “super-aged” societal classifications. Although heart failure (HF) can occur in all age groups, it is primarily a disease of the elderly, with increased prevalence in older people. The increasing burden of HF with global aging is a socioeconomic problem worldwide, especially in aged and super-aged societies. Furthermore, advances in the treatment and prevention of ischemic heart disease has also prolonged the lives of patients with heart disease, further contributing to the rise in HF prevalence.

Patients with HF are getting older, and physicians need to be delicate in treating these elderly patients, as they are known to have a poor prognosis [1] and there is a gap in evidence when prescribing guideline-directed medical therapies (GDMT) in this population. Large-scaled, randomized clinical trials that have incorporated renin–angiotensin system (RAS) inhibitors, beta-blockers, and aldosterone antagonists (AA) into guidelines have been mostly performed on patients in their mid-60s [2,3,4,5]. Although trials such as SENIORS (Study of Effects of Nebivolol Intervention on Outcomes and Rehospitalization in Seniors With Heart Failure) [6], I-PRESERVE (Irbesartan in Heart Failure With Preserved Ejection Fraction) [7], and PEP-CHF (Perindopril in Elderly People With Chronic Heart Failure) [8] have expanded the age limit and compared treatments in patients aged ≥70 years, real-world HF patients are still different from ideal study candidates [9,10].

The current study aimed to help reduce the gap regarding patient management between clinical trials and real-world practice, as well as to provide evidence for the adequate treatment of elderly HF patients using data from a large-scaled, long-term follow-up acute HF registry.

## 2. Experimental Section

### 2.1. Study Population

The Korean Acute Heart Failure (KorAHF) registry is a prospective, multi-center study designed to assess clinical features and outcomes of acute HF in Korea. We enrolled patients hospitalized for acute HF in 10 nationwide centers from March 2011 to February 2019. Detailed information on the design and interim results of the KorAHF registry have been previously published [11,12]. Accurate data on mortality was acquired using information from the National Health Insurance Service or government-reported death records. All enrolled patients were referred to a cardiologist specializing in HF management, and optimal medical and device therapy was encouraged at the discretion of the treating physician. Clinical events were monitored and verified by a separate committee composed of independent experts who had not participated in patient enrollment [11]. Prescription of GDMT was investigated at discharge and at each follow-up visit, which was scheduled at <1, 3, 6, 12, 24, 36, 48, and 60 months after discharge. The study was conducted in accordance with the Declaration of Helsinki, and written informed consent was obtained before enrollment. All study protocols were reviewed and approved by the Institutional Review Board of each participating hospital, and also registered with ClinicalTrials.gov (NCT01389843).

### 2.2. Outcome Measures

Patients were enrolled on admission of an acute HF event, and those surviving to discharge without heart transplantations were included in the current analysis. We defined the elderly as those aged ≥80 years (octogenarians) at time of enrollment. Long-term all-cause mortality was assessed at 5 years, and mortality and readmissions for worsening HF were additionally assessed at 6 months. Outcomes were compared between elderly and non-elderly patients in the total study population, and the association of medications and outcomes were additionally assessed in octogenarians with reduced and preserved ejection fractions (EF). Target doses of GDMT were defined following the 2016 European Society of Cardiology (ESC) Guidelines for the diagnosis and treatment of acute and chronic heart failure [13].

### 2.3. Statistical Analyses

Continuous variables are expressed as mean ± standard deviation or median with interquartile range (IQR), and were compared using the independent *t*-test, analysis of variance (ANOVA), or Mann–Whitney U test. Categorical variables are given as numbers and percentages and were compared using the chi-squared test. Blood pressures (BP) were measured at each predefined follow-up visit, and on-treatment BPs were calculated by averaging measurements before the occurrence of an event or end of follow-up [14]. Restricted cubic splines and nonlinear Cox proportional hazard regression models were used to assess the association between BP and outcomes.

Presence of sarcopenia was retrospectively assessed using the skeletal muscle mass index (SMI), a ratio of skeletal muscle to total body mass. Skeletal muscle mass was acquired using a “body-weight and height model” equation as follows:

Skeletal mass (kg) = 0.244 × body weight + 7.8 × height − 0.098 × age + 6.6 × sex + race − 3.3(1)

(sex = 1 for male, 0 for female, and race = −1.2 for Asians) [15]. Previously reported cutoff values for SMI were used to assess presence of sarcopenia [16]. Event rates were estimated using the Kaplan–Meier method and the log-rank test was used to compare between treatments. To adjust for changes in medications during the early follow-up period, landmark analysis was additionally performed at 6 months when comparing between treatments. Multivariate Cox proportional hazard regression models were constructed using age, sex, previous HF, hypertension, diabetes, EF, serum hemoglobin, serum creatinine, and use of GDMT as covariates. All analyses were performed using SPSS version 25 (IBM Crop., Armonk, NY, USA) and R programming 3.6.0 (http://www.R-project.org; The R Foundation, Vienna, Austria). All *p*-values are two-sided, and *p*-values < 0.05 are considered to be significant.

## 3. Results

### 3.1. Patient Characteristics

Among the 5625 patients enrolled in the KorAHF registry, 5293 patients survived to discharge after a median of 9 (IQR 6–14) hospital days. Mean patient age was 68.6 ± 14.3 years, 52.8% were male, and 55.2% had an EF of less than 40% at admission. At time of enrollment, 1185 (22.4%) patients were aged ≥80 years, and significant differences were observed in baseline characteristics compared with non-elderly patients. They were more likely to be female, have an ischemic etiology of HF, have preserved left ventricular systolic function, and present with a lower body mass index (BMI) and hemoglobin levels, while levels of natriuretic peptides were increased compared with the non-elderly population. The prevalence of sarcopenia was also higher in octogenarians. The complete baseline demographics of study participants are shown in Table 1 according to age group.

### 3.2. Characteristics of Octogenarian HF Patients

**Figure 1:**
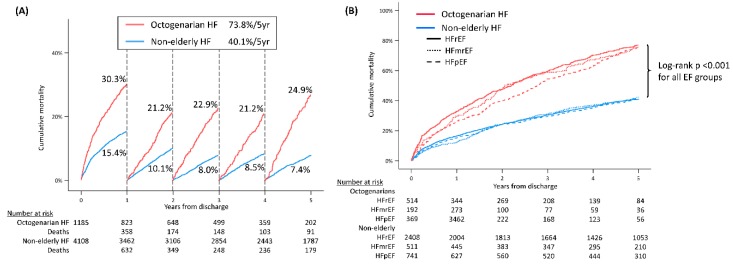
Prognosis of octogenarian HF. (**A**) Annual mortality of octogenarian patients compared with non-elderly patients. (**B**) All-cause mortality according to age and EF category. HF, heart failure; EF, ejection fraction; HFrEF, HF and reduced EF; HFmrEF, HF and mid-range EF; HFpEF, HF and preserved EF.

During a median follow-up of 4.3 years (IQR 1.6–5.6 years), elderly patients showed a significantly higher rate of overall mortality compared with the non-elderly (29.29 per 100 person-years vs. 11.13 per 100 person-years, *p* < 0.001). Kaplan–Meier survival curves showed continuous divergence of the octogenarian and the non-elderly patients evident from the early follow-up periods (Figure 1A), regardless of EF (Figure 1B). According to multivariate Cox proportional hazard regression models, old age (age ≥ 80) was a significant predictor for both all-cause mortality (HR (hazard ratio) 1.93, 95% CI 1.76–2.11, *p* < 0.001) and readmissions for worsening HF (HR 1.27, 95% CI 1.13–1.43, *p* < 0.001). In octogenarians, male sex was an independent risk of all-cause mortality (HR 1.19, 95% CI 1.01–1.40, *p* = 0.034), whereas diabetes was a significant predictor in the non-elderly. Sarcopenia was a common risk factor both age groups. Multivariate Cox models for mortality and HF readmissions according to age are described in Tables S1–S4.

### 3.3. Blood Pressure and Clinical Outcomes in Octogenarians

**Figure 2:**
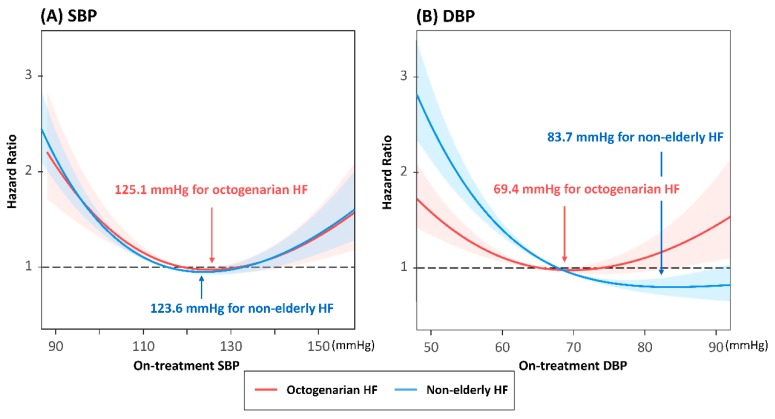
Restricted cubic splines for all-cause mortality according to on-treatment (**A**) SBP and (**B**) DBP. SBP, systolic blood pressure; DBP, diastolic blood pressure; HF, heart failure.

Restricted cubic splines were drawn using significant covariates derived from Cox models described in a previous publication [13]. As shown in Figure 2, a J-curve association was observed between on-treatment BP and all-cause mortality, with risk increasing at both low and high BP values. According to nonlinear Cox regression analysis, the nadir BP value correlating to lowest risk was 125.1 mmHg for systolic blood pressure (SBP; chi-square 69.8, degrees of freedom (df) = 2, *p* < 0.001) and 69.4 mmHg for diastolic blood pressure (DBP; chi-square 12.1, df = 2, *p* < 0.001). The non-linear association between on-treatment BP and mortality was similar in both elderly and non-elderly patients, but the nadir DBP was lower in octogenarians (69.4 mmHg vs. 83.7 mmHg). The association between DBP and outcome was also more U-shaped in octogenarians, with risk also increasing at higher values compared with non-elderly patients. The risk for mortality according to each BP category is shown in Figure S1.

### 3.4. Impact of GDMT in Octogenarians with HF and Reduced EF

Octogenarian patients with HF and reduced EF (HFrEF) were less likely to receive GDMT compared with non-elderly patients. The prescription rates of RAS inhibitors (74.3% vs. 78.4%, *p* = 0.041), beta-blockers (47.1% vs. 59.8%, *p* < 0.001), and AAs (46.1% vs. 55.2%, *p* < 0.001) at discharge were lower in octogenarians (Figure S2). During follow-up, prescription rates for RAS inhibitors and AAs further decreased, whereas that of beta-blockers showed an increase during the first year. The proportion of patients receiving adequate doses were also low for RAS inhibitors and beta-blockers, with only 27.8% and 10.0% of patients receiving at least half the target dose, respectively (Table 1).

**Figure 3:**
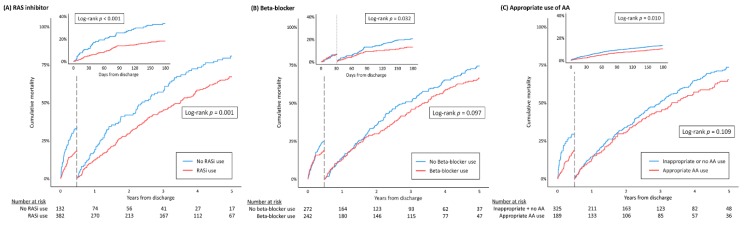
Kaplan–Meier curves for all-cause mortality according to guideline-directed medical therapies (GDMT). (**A**) RAS inhibitor; (**B**) beta-blocker; (**C**) appropriate use of AA. GDMT, guideline-directed medical therapy; RAS, renin angiotensin system; AA, aldosterone antagonist.

For octogenarian HFrEF patients, the all-cause mortality rate was significantly lower in those using RAS inhibitors (64.2% vs. 79.2% at 5 years, 18.1% vs. 33.3% at 6 months for users and non-users, respectively) (Figure 3A). In covariate-adjusted Cox proportional hazard regression models, RAS inhibitor use was associated with a significantly lower incidence of all-cause mortality (HR 0.77, 95% CI 0.61–0.98, *p* = 0.031), but not with readmissions for worsening HF (HR 0.75, 95% CI 0.70–1.38, *p* = 0.777).

**Figure 4:**
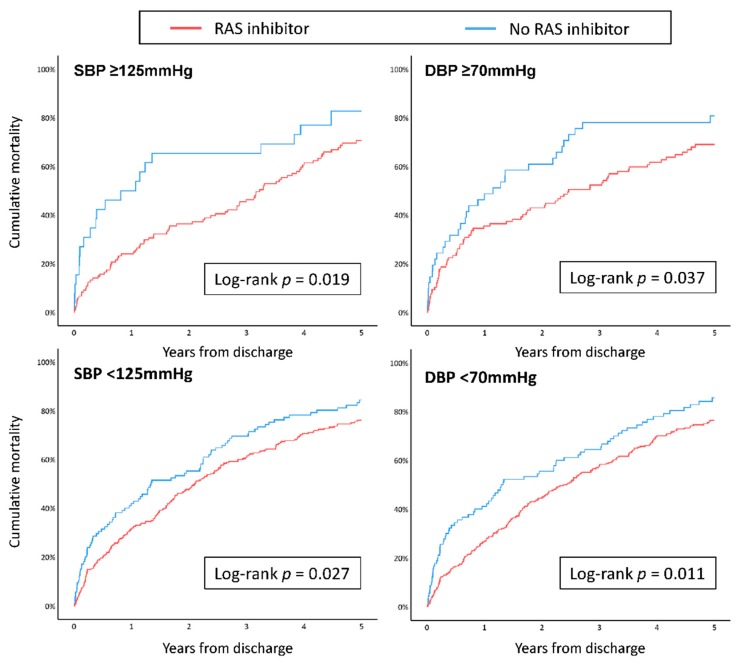
Kaplan–Meier curves for all-cause mortality according to BP and RAS inhibitor use. BP, blood pressure; RAS, renin–angiotensin system; SBP, systolic blood pressure; DBP, diastolic blood pressure.

The effect of RAS inhibitor use on outcomes were further compared using BP values at nadir points acquired from previous nonlinear Cox regression analyses (125 mmHg for SBP, and 70 mmHg for DBP). The proportion of patients using RAS inhibitors were lower in patients with SBP < 125 mmHg (71.1% vs. 82.3%, *p* = 0.009), whereas prescription of AAs (49.0% vs. 38.8%, *p* = 0.035) and loop diuretics (81.7% vs. 64.6%, *p* < 0.001) were increased. For DBP, there were no significant differences except for diuretics, which showed increased use in patients with DBP < 70 mmHg (79.6% vs. 70.7%, *p* = 0.028). However, use of RAS inhibitors was also associated with significantly lower mortality rates even at lower systolic and diastolic BP (Figure 4). For beta-blockers, there were no significant differences in prescription rates according to BP (46.0% vs. 49.7%, *p* = 0.459 for SBP 125 mmHg; 45.7% vs. 50.3%, *p* = 0.330 for DBP 70 mmHg). Beta-blockers were associated with numerically lower, but non-significant, mortality rates at lower BPs (Figure S3).

The prescription rate of beta-blockers in octogenarian patients with HFrEF were less than 50%, and only 10% of users were receiving at least 50% of the recommended target dose. In contrast to younger patients, beta-blocker use was not a significant predictor for 5 year all-cause mortality (HR 0.87, 95% CI 0.71–1.06, *p* = 0.167) in octogenarians. However, after adjusting for early post-discharge mortality and changes in medication, use of beta-blockers was associated with a significantly lower 6 month mortality compared with those without treatment (10.8% vs. 20.9%, HR 0.52, 95% CI 0.32–0.86, *p* = 0.011). Beyond 6 months, beta-blocker therapy was still associated with a non-significant, but numerically lower rate of mortality (64.6% vs. 73.1%, log-rank *p* = 0.097; Figure 3B).

Among GDMTs, AAs had the lowest prescription rate in octogenarian patients. In addition, AAs were being used inappropriately in patients with poor renal function. Only 41.6% of octogenarian HFrEF patients who would have benefited from AAs were actually using them, whereas 15.4% of users showed decreased renal function (serum creatinine > 2.5 mg/dL) and/or elevated potassium levels (serum potassium > 5.0 mmol/L). There was no difference in mortality rates according to AA use at discharge (Figure S4), but a significantly lower mortality rate was observed at 6 months in patients who were using AAs as indicated (18.5% vs. 28.8%, log-rank *p* = 0.047), as shown in Figure 3C. For readmissions due to worsening HF, use of GDMT was associated with a lower incidence of events overall, but the difference was not significant (Figure S5, Table S5).

### 3.5. RAS Inhibitors in Octogenarians with HF and Preserved EF

**Figure 5:**
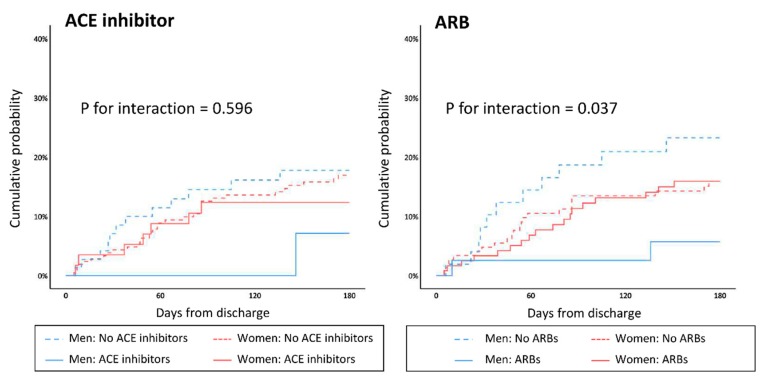
Sex difference in HF readmissions according to use of ACE inhibitors and ARBs. HF, heart failure; ACE, angiotensin-converting enzyme; ARB, angiotensin receptor blocker.

For octogenarians with HF and preserved EF (HFpEF), RAS inhibitor use was associated with a significantly lower rate of HF readmissions at 6 months (log-rank *p* = 0.035). The difference was mostly driven by the difference in males (HR 0.15, 95% CI 0.04–0.58, log-rank *p* = 0.001), whereas only a modest, non-significant difference was observed in female patients (HR 0.81, 95% CI 0.41–1.60, log-rank *p* = 0.558). When RAS inhibitor use in males was further divided as use of angiotensin-converting enzyme (ACE) inhibitors or angiotensin receptor blockers (ARB), ACE inhibitors showed a non-significant tendency toward lower HF readmissions (HR 0.34, 95% CI 0.04–2.77), whereas the significant benefit persisted with ARBs (HR 0.19, 95% CI 0.04–0.87), as shown in Figure 5. This sex difference in HFpEF patients was not observed in mortality, nor in non-elderly patients.

## 4. Discussion

In this analysis of octogenarian HF patients using a dedicated long-term registry, we were able to show that medications known to improve outcomes are not prescribed sufficiently in real-world practice. Even so, octogenarian patients who did receive adequate treatment showed significant reduction in mortality. Not many studies have focused on treatment of super-aged HF patients, and we believe that our results supply valuable information to remind physicians that GDMT is also important in elderly HF patients.

Results from randomized clinical trials are regarded as the highest level of evidence in current medicine. However, not all results can be readily applied to daily practice, as the ideal study population does not fully reflect real-world patients due to strict inclusion and exclusion criteria. In the KorAHF registry, we enrolled over 5000 acute HF patients without any specific exclusion criteria, and were able to reflect patients being treated in the clinic every day. Furthermore, one of the strengths of this registry is that we were able to acquire accurate data on mortality owing to a centralized, government-run insurance policy. Even with intrinsic limitations that registry data face, we believe that results of our analysis will clarify areas where randomized trials have not been so clear.

### 4.1. Distinctions of Octogenarian HF Patients

The octogenarian HF population was vastly different, not only in clinical presentation, but also in prognostic factors and outcomes. As old age is a significant predictor of poor outcome regardless of HF, it is not surprising that octogenarian patients had worse outcomes compared with their younger counterparts. However, presence of HF in octogenarians nearly doubled the mortality rate, compared to the general Korean octogenarian population (73.8% vs. 42.9% for 5 year mortality) [17]. In a study analyzing long-term mortality after coronary artery bypass grafting, octogenarians showed a mortality rate of 23% 5 years post-surgery [18], and a 5 year mortality rate of 44% has been reported in octogenarians undergoing surgical aortic valve replacement [19], which is lower than the mortality seen in our cohort of HF patients. A sex difference in prognosis was also observed in octogenarian HF patients, similar to that which has also been observed in European patients [20]. Sarcopenia, which is closely related with frailty, was found to be more prevalent in the elderly, and was an independent predictor of mortality. Finally, the prescription rates of medications with known survival benefits were lower than non-elderly patients.

### 4.2. Use of GDMT

In our study cohort, octogenarian patients with HFrEF were not receiving adequate GDMT, even though all patients were followed-up by HF specialists. Inadequate use of medications proven to improve clinical outcomes have been reported repeatedly, and this is not a problem that only affects octogenarians. Recent analysis from the CHAMP-HF (Change the Management of Patients with Heart Failure) registry showed that there were significant gaps in use and doses of GDMT in HFrEF patients [21]. Under-treatment in elderly patients has also been previously reported in various studies [22,23]. The reason for the lower prescription rate probably lies on the many obstacles that need to be overcome in prescribing GDMT to the elderly. First of all, BP in elderly HF patients, especially those with reduced EF, may not be adequate enough to allow physicians to prescribe medications that also have BP-lowering effects. Even after taking into account that the elderly may require lower doses due to being frailer and having lower BMI [24], only 27.8% and 10% of octogenarian HFrEF patients received at least half the target dose of RAS inhibitors and beta-blockers, respectively. The fact that a majority of patients could not tolerate the recommended dose has also been reported in previous studies [22,25], adding to the evidence that controlled trials and real-world practice are different.

However, the nadir point for DBP associated with mortality was lower in octogenarians, suggesting that they might be more tolerable to lower BP than relatively younger patients. Even for patients with SBP < 125 mmHg or DBP < 70 mmHg, use of RAS inhibitors was associated with a significant reduction in mortality. Octogenarian patients may be chronically adapted to lower BP and less prone to perform abrupt postural changes, which reduces the likelihood of postural dizziness or syncope, and makes them more tolerable to medications. Although the mechanism is not clear, the SPRINT subgroup analysis has also reported that older hypertensive patients showed more favorable outcomes with intensive BP-lowering treatments [26]. Even with lower doses than the guidelines recommend, those prescribed with RAS inhibitors and beta-blockers were still associated with improved survival, suggesting that target doses of GDMT may need to be adjusted according to ethnicity, as East Asians may have lower body weights and BMI compared to Western patients. Physicians should be more resilient in starting necessary medications, and physician inertia should not be the reason medications known to improve outcomes are withheld from elderly patients with marginal BP.

Few studies have evaluated the association between GDMT use and prognosis in elderly HF patients, and have produced inconsistent results. Use of RAS inhibitors have shown to improve survival in octogenarians in the EHFS II (EuroHeart Failure Survey II) [27], and beta-blockers have been proven to be beneficial in patients ≥70 years in the SENIORS trial [6]. On the other hand, GBMT had no effect on survival in an analysis of the WET-HF (West Tokyo Heart Failure) registry [28]. These conflicting results may be due to differences in patient characteristics according to region, or by increased use of newer generation beta-blockers such as carvedilol or nebivolol in recent years. Further studies would be needed to firmly recommend appropriate GDMT use in the elderly population.

### 4.3. Octogenarians with HFpEF

For HFpEF patients, there are still no definite guideline-recommended medications known to improve survival. Recent studies have advocated the possibility of angiotensin receptor/neprilysin inhibitors (ARNI) or sodium-glucose transport 2 (SGLT2) inhibitors, but further studies are still warranted. In our analysis of octogenarian HFpEF patients, use of RAS inhibitors, and especially ARBs, were associated with a significant reduction in HF readmissions at 6 months. Of further interest is that a sex difference was observed, and that men with HFpEF benefited from using ARBs. A recent report from PARAGON-HF (Prospective Comparison of ARNI With ARB Global Outcomes in HF With Preserved Ejection Fraction) showed a significant reduction in hospitalizations among women using sacubitril/valsartan [29]. As the comparator group was using valsartan, an ARB, it has been suggested that this result rise from an increased effect of ARB in men. The difference in effect of ARBs or ACE inhibitors could be explained by the fact that the men in our study, compared to women, had a higher number of patients with an ischemic etiology (31.6% vs. 18.7%, *p* = 0.009). This has also been described in the HOPE (Heart Outcomes Prevention Evaluation) study, which suggested that benefits of RAS inhibitors were due in part to a vasculoprotective effect [2]. Sex differences in HFpEF treatment has also been seen in the case of spironolactone, which showed a more favorable outcome in women [30]. Future studies would be needed to see if there is a subgroup of HFpEF patients who would benefit from certain therapies.

Kaplan–Meier curves for mortality according to use of beta-blockers diverged in the early periods for non-elderly patients, but curves for elderly patients saw no such difference up to 30 days post-discharge. The survival benefit of beta blockade was more evident in patients surviving the first month after discharge. This could be explained by the fact that the rate of early mortality is especially high in elderly patients [31], and that beta-blockers were added on during the early follow-up periods. A previous study in chronic HF patients also reported that a quarter of admitted HF patients stopped beta-blockers or reduced their dosage at discharge due to hypotension of use of inotropes [32].

The last important observation is that AAs were not being properly used in elderly HF patients. On the basis of the study population in RALES (Randomized Aldactone Evaluation Study) [5] and EMPHASIS-HF (Eplerenone in Mild Patients Hospitalization and Survival Study in Heart Failure) [33], guidelines suggest that AAs should be used in symptomatic HFrEF patients without significant renal dysfunction or hyperkalemia [13]. However, in our study cohort, patients with poor renal function and/or hyperkalemia were still receiving treatment, whereas over 40% of those who should be using AAs were not. When AA use was compared in patients who were properly using them, we were able to observe a survival gain in elderly patients with HFrEF, suggesting that inadequate use of AAs could have harmful effects on elderly patients with marginal renal function.

### 4.4. Limitations

The limitations of the study are as follows. First, the current study used data from an observational registry. Although efforts were taken to adjust for differences in baseline characteristics, there are limitations that are intrinsic in the design. Second, the prescription of medications was not fixed during follow-up. Patients may have discontinued or initiated certain treatments during follow-up. We tried to assess the changes in medications by performing separate analyses adjusting for changes in medications up to 6 months, but discontinuations and initiations afterwards could have affected results. Third, as mentioned above, increased age is a significant risk for mortality and morbidity, and the effect of hidden factors could have influenced outcomes. As long-term outcomes were more likely to be influenced by old age and frailty, we assessed mid-term outcomes at 6 months, and also performed landmark analysis at 30 days to exclude severely ill patients. Furthermore, although age is a continuous variable, it was dichotomized to emphasize the unique characteristics of elderly patients. Finally, the number of patients used in our analyses might not have provided sufficient power to fully support our findings. Well-designed, prospective randomized controlled trials focusing on elderly HF patients would be needed to fully understand the complexity in treating these patients.

## 5. Conclusions

HF in octogenarians have different characteristics compared with the non-elderly. However, adequate use of GDMT was still associated with improved survival. Physicians should be more vigilant in prescribing medications with clinical benefits, even in elderly patients with HF.

## Figures and Tables

**Table 1:** Baseline characteristics.

	Total (*n* = 5293)	Octogenarian HF (*n* = 1185)	Non-Elderly HF (*n* = 4108)	*p*
**Demographics**				
Age (years) (min, max)	68.6 ± 14.3 (18,112)	84.6 ± 4.0 (80,112)	64.0 ± 12.8 (18,79)	<0.001
Male	2794 (52.8)	432 (36.5)	2362 (57.5)	<0.001
BMI (kg/m^2^)	23.4 ± 3.9	22.2 ± 3.6	23.7 ± 3.9	<0.001
Current smoking	934 (17.6)	88 (7.4)	846 (20.6)	<0.001
**Comorbidities**				
Hypertension	3315 (62.6)	899 (75.9)	2416 (58.8)	<0.001
Diabetes	2079 (39.3)	412 (34.8)	1667 (40.6)	<0.001
Previous HF	2504 (47.3)	602 (50.8)	1902 (46.3)	0.007
Atrial fibrillation	1520 (28.7)	377 (31.8)	1143 (27.8)	0.008
COPD	586 (11.1)	167 (14.1)	419 (10.2)	<0.001
Sarcopenia	2135 (40.7)	731 (62.5)	1404 (34.4)	<0.001
SMI (%)	6.1 ± 1.3	5.1 ± 1.0	6.4 ± 1.2	<0.001
**Etiology**				
Ischemic	1955 (36.9)	489 (41.3)	1466 (35.7)	0.001
Idiopathic DCM	1092 (20.6)	155 (13.1)	937 (22.8)	<0.001
**Clinical status**				
NYHA III-IV at discharge	557 (10.5)	182 (15.8)	375 (9.4)	<0.001
On-treatment SBP	115.9 ± 16.0	118.9 ± 15.9	115.0 ± 16.0	<0.001
On-treatment DBP	67.6 ± 9.7	65.9 ± 9.8	68.0 ± 9.7	<0.001
On-treatment heart rate	78.1 ± 13.0	78.3 ± 13.5	78.1 ± 12.8	0.613
Ejection fraction (%)	38.0 ± 15.5	42.3 ± 15.0	36.8 ± 15.4	<0.001
HFpEF (EF > 50%)	1109 (23.4)	368 (34.3)	741 (20.2)	<0.001
**Laboratory**				
Hemoglobin (g/dL)	12.4 ± 2.3	11.7 ± 1.9	12.6 ± 2.4	<0.001
Potassium (mmol/L)	4.4 ± 0.7	4.4 ± 0.7	4.4 ± 0.7	0.970
eGFR (mL/min/1.73 m^2^)	57.9 ± 35.9	36.3 ± 16.1	64.1 ± 37.6	<0.001
BNP (pg/mL)	883.5 (464.0, 1700.7)	993.5 (527.6, 1830.6)	846.0 (451.0, 1630.0)	0.003
NT-proBNP (pg/mL)	4737 (2086,11389)	6257 (2776,14528)	4374 (1944,10384)	<0.001
**Hospital course**				
IV inotropes	1457 (27.5)	260 (21.9)	1197 (29.1)	<0.001
IV nitrates	2133 (40.3)	569 (48.0)	1564 (38.1)	<0.001
Hospital stay (days)	9.0 (6.0, 14.0)	8.0 (6.0, 13.0)	9.0 (6.0, 15.0)	0.002
**Medication at discharge**				
RAS inhibitor	3683 (69.6)	816 (68.9)	2867 (69.8)	0.540
≥50% of target dose	1060 (28.5)	227/816 (27.8)	823/2867 (28.7)	0.620
ACE inhibitor	1581 (29.9)	324 (27.3)	1257 (30.6)	0.031
ARB	2127 (40.2)	495 (41.8)	1632 (39.7)	0.206
Beta blocker	2779 (52.5)	529 (44.6)	2250 (54.8)	<0.001
≥50% of target dose	292/2779 (10.5)	53/529 (10.0)	239/2250 (10.6)	0.714
AA	2491 (47.1)	504 (42.5)	1987 (48.4)	<0.001
≥50% of target dose	1562 (62.7)	298/504 (59.1)	1264/1987 (63.6)	0.063
Loop diuretic	3861 (72.9)	863 (72.8)	2998 (73.0)	0.917

HF, heart failure; BMI, body mass index; COPD, chronic pulmonary obstructive disease; SMI, skeletal muscle mass index; DCM, dilated cardiomyopathy; NYHA, New York Heart Association functional class; SBP, systolic blood pressure; DBP, diastolic blood pressure; HFpEF, HF and preserved ejection fraction; eGFR; estimated glomerular filtration rate; BNP, brain natriuretic peptide; NT-proBNP, N-terminal pro-BNP; RAS, renin–angiotensin system; ACE, angiotensin-converting enzyme; ARB, angiotensin receptor blocker; AA, aldosterone antagonist.

## Supplementary Materials

The following are available online at https://www.mdpi.com/2077-0383/9/2/501/s1: Figure S1: Risk of mortality according to blood pressure categories. Figure S2: Prescription rates of GDMT according to age and EF category. Figure S3: Kaplan–Meier curves for all-cause mortality according to BP and beta-blocker use. Figure S4: Kaplan–Meier curve for cumulative all-cause mortality according to use of aldosterone antagonists at discharge in octogenarians with HFrEF. Figure S5: Kaplan–Meier curves for 6 month HF readmissions according to use of GDMT in octogenarians with HFrEF. Table S1: Multivariable Cox regression model for all-cause mortality in octogenarian patients with HF. Table S2: Multivariable Cox regression model for all-cause mortality in non-elderly patients with HF. Table S3: Multivariable Cox regression model for HF readmissions in octogenarian patients. Table S4: Multivariable Cox regression model for HF readmissions in non-elderly patients. Table S5: Adjusted hazard ratios for all-cause mortality and HF readmissions by use of GDMT in octogenarian patients with HFrEF.
