# Supplementary material for: Management and Prognosis of Heart Failure in Octogenarians: Final Report from the KorAHF Registry
Source: J Clin Med. 2020 Feb 12;9(2):501. doi: 10.3390/jcm9020501 (PMC7074088; doi:10.3390/jcm9020501)
Supplement: Supplementary file 1 [file jcm-09-00501-s001.pdf]

Journal of Clinical Medicine

Article

Management and Prognosis of Heart Failure in Octogenarians: Final report from the KorAHF Registry

Gyu Chul Oh [1], Hyun-Jai Cho [1], Sang Eun Lee [2], Min-Seok Kim [2], Jae-Joong Kim [2], Jin-Oh Choi [3], Eun-Seok Jeon [3], Kyung-Kuk Hwang [4], Shung Chull Chae [5], Sang Hong Baek [6], Seok-Min Kang [7], Byung-Su Yoo [8], Dong-Ju Choi [9], Youngkeun Ahn [10], Kye Hun Kim [10], Myeong-Chan Cho [4], Byung-Hee Oh [1] and Hae-Young Lee [1,*]

1. Supplementary Tables

Table 1. Multivariable Cox regression model for all-cause mortality in octogenarian patients with HF.

	Adjusted HR (95% CI)	*p*-Value
Age (for every 1-year increase)	1.05 (1.04–1.07)	<0.001
Sex (Male)	1.19 (1.01–1.40)	0.034
Previous HF	1.22 (1.07–1.40)	0.004
Hemoglobin < 12 g/dL	1.47 (1.28–1.69)	<0.001
EF (for every 5% decrease)	1.04 (1.02–1.06)	0.002
Creatinine (for every 1 mg/dL increase)	1.06 (1.01–1.12)	0.033
Sarcopenia	1.28 (1.09–1.51)	0.003
Use of RAS inhibitors	0.86 (0.74–1.00)	0.046
Use of beta-blockers	0.94 (0.82–1.08)	0.414
Use of AAs	1.02 (0.89–1.18)	0.743

HF, heart failure; HR, hazard ratio; CI, confidence interval; EF, ejection fraction; RAS, renin-angiotensin aldosterone system; AA, aldosterone antagonist.

Table 2. Multivariable Cox regression model for all-cause mortality in non-elderly patients with HF.

	Adjusted HR (95% CI)	*p*-Value
Age (for every 1-year increase)	1.04 (1.03–1.04)	<0.001
Previous HF	1.70 (1.54–1.89)	<0.001
Diabetes	1.25 (1.13–1.38)	<0.001
Hemoglobin < 12 g/dL	1.70 (1.53–1.88)	<0.001
EF (for every 5% decrease)	1.05 (1.03–1.07)	<0.001
Creatinine (for every 1 mg/dL increase)	1.07 (1.04–1.10)	<0.001
Sarcopenia	1.47 (1.33–1.63)	<0.001
Use of RAS inhibitors	0.79 (0.71–0.88)	<0.001
Use of beta-blockers	0.77 (0.70–0.85)	<0.001
Use of AAs	1.08 (0.98–1.20)	0.125

HF, heart failure; HR, hazard ratio; CI, confidence interval; EF, ejection fraction; RAS, renin-angiotensin aldosterone system; AA, aldosterone antagonist.

Table 3. Multivariable Cox regression model for HF readmissions in octogenarian patients.

	Adjusted HR (95% CI)	*p*-Value
Previous HF	1.85 (1.38–2.48)	<0.001
EF (for every 5% decrease)	1.04 (1.00–1.12)	0.077
Use of RAS inhibitors	0.71 (0.53–0.95)	0.022
Use of beta-blockers	0.78 (0.59–1.04)	0.091
Use of AAs	0.98 (0.74–1.29)	0.876

HF, heart failure; HR, hazard ratio; CI, confidence interval; EF, ejection fraction; RAS, renin-angiotensin aldosterone system; AA, aldosterone antagonist.

Table 4. Multivariable Cox regression model for HF readmissions in non-elderly patients.

	Adjusted HR (95% CI)	*p*-Value
Age (for every 1-year increase)	1.01 (1.00–1.02)	0.002
Previous HF	2.16 (1.82–2.57)	<0.001
Diabetes	1.31 (1.11–1.54)	0.001
Hemoglobin <12 g/dL	1.22 (1.04–1.45)	0.018
EF (for every 5% decrease)	1.04 (1.02–1.07)	0.001
Use of RAS inhibitors	0.87 (0.72–1.03)	0.109
Use of beta-blockers	0.67 (0.57–0.79)	<0.001
Use of AAs	1.07 (0.91–1.26)	0.393

HF, heart failure; HR, hazard ratio; CI, confidence interval; EF, ejection fraction; RAS, renin-angiotensin aldosterone system; AA, aldosterone antagonist.

Table S5. Adjusted hazard ratios for all-cause mortality and HF readmissions by use of GDMT in octogenarian patients with HFrEF.

	All-Cause Mortality		*Readmission for Worsening HF*	
	**Adjusted HR (95% CI)**	***p*-Value**	**Adjusted HR (95% CI)**	***p*-Value**
RAS inhibitors	0.77 (0.61–0.98)	0.031	0.75 (0.70–1.38)	0.777
Beta-blockers	0.90 (0.73–1.11)	0.332	0.63 (0.45–0.86)	0.004
AAs	1.03 (0.83–1.28)	0.788	1.15 (0.82–1.60)	0.423

GDMT, guideline-directed medical therapy; HR, hazard ratio; CI, confidence interval.

2. Supplementary Figures

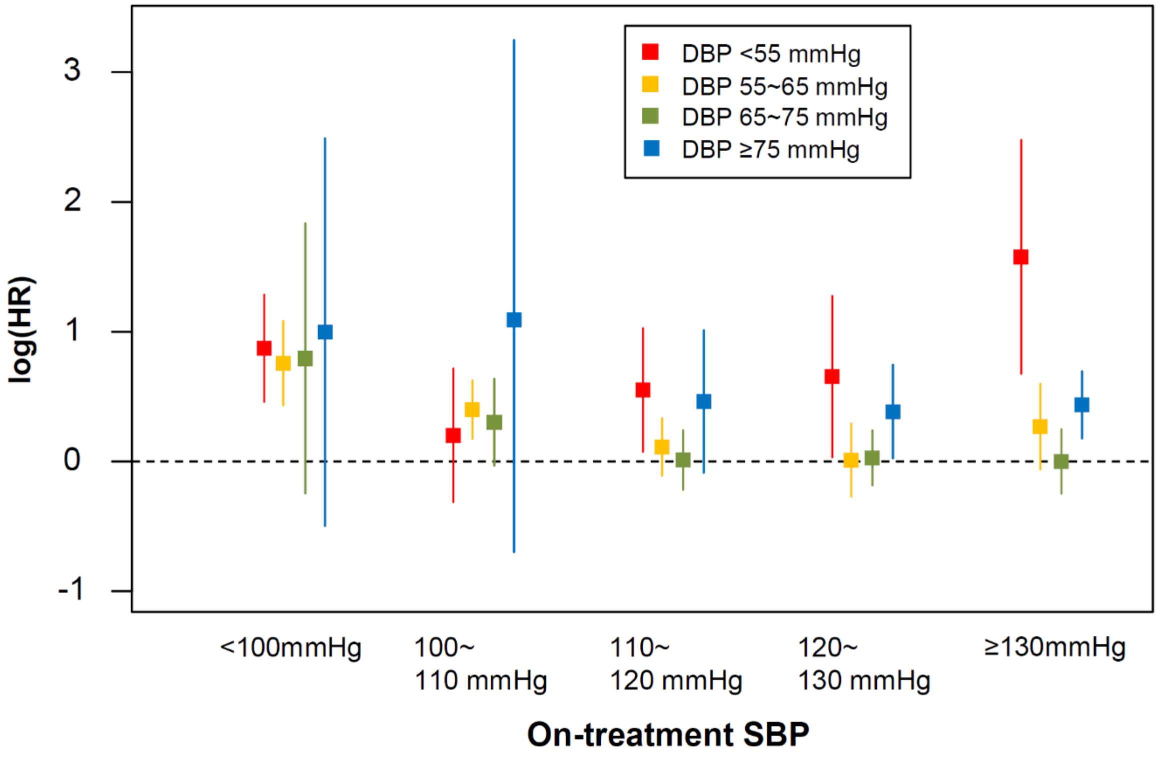

Figure S1. Risk of mortality according to blood pressure categories. HR, hazard ratio; SBP, systolic blood pressure; DBP, diastolic blood pressure.

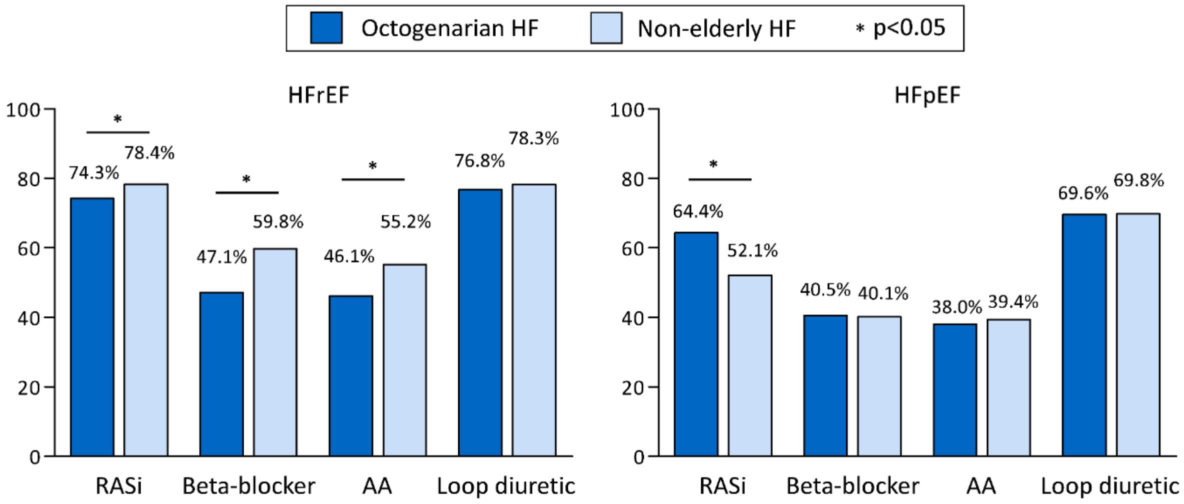

Figure S2. Prescription rates of GDMT according to age and EF category. GDMT, guideline directed medical therapy; EF, ejection fraction; HF, heart failure; HFrEF, HF with reduced EF; HFpEF, HF with preserved EF; RASi, renin-angiotensin system inhibitor; AA, aldosterone antagonist.

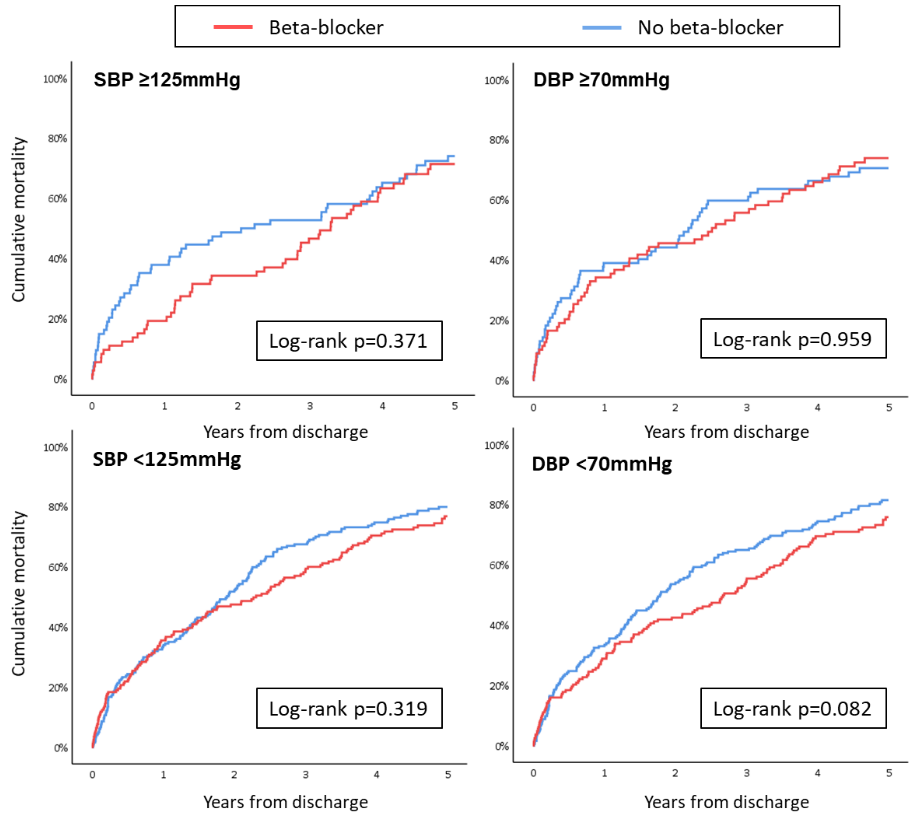

Figure S3. Kaplan-Meier curves for all-cause mortality according to BP and beta-blocker use. BP, blood pressure; SBP, systolic blood pressure; DBP, diastolic blood pressure.

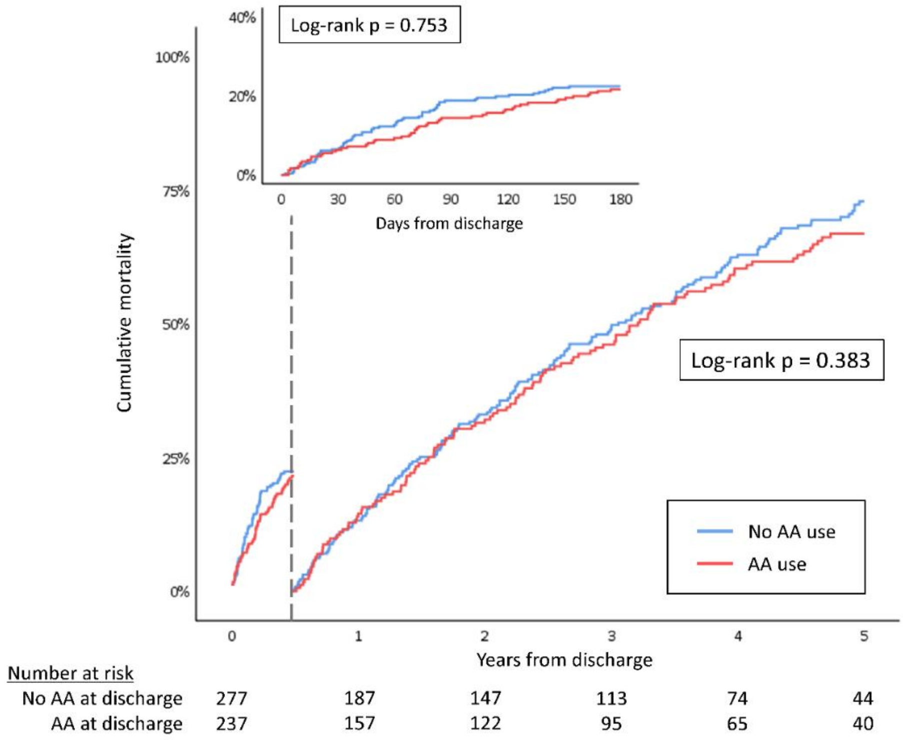

Figure S4. Kaplan-Meier curve for cumulative all-cause mortality according to use of aldosterone antagonists at discharge in octogenarians with HFrEF. AA, aldosterone antagonist; HFrEF, heart failure and reduced ejection fraction.

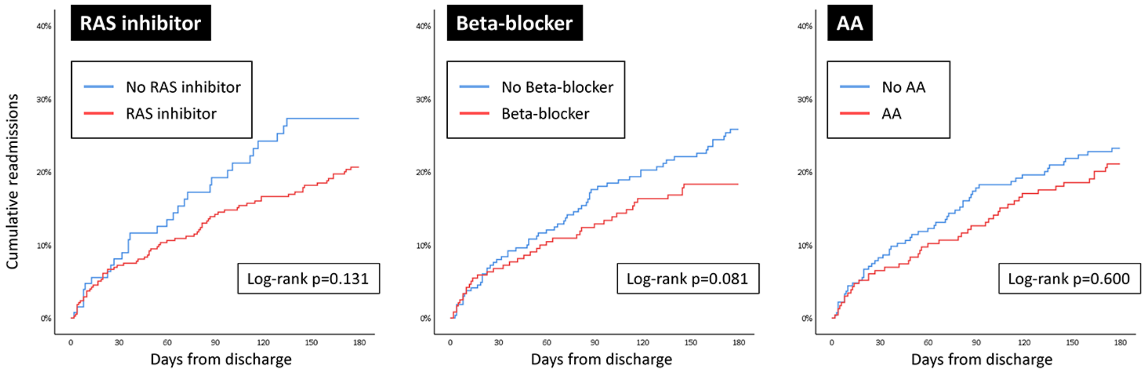

Figure S5. Kaplan-Meier curve for 6-month HF readmissions according to use of GDMT in octogenarians with HFrEF. HF, heart failure; GDMT, guideline directed medical therapy; HFrEF, heart failure and reduced ejection fraction; RAS, renin-angiotensin system; AA, aldosterone antagonist.
